# Pituitary Pathology and Gene Expression in Acromegalic Cats

**DOI:** 10.1210/js.2018-00226

**Published:** 2018-10-16

**Authors:** Christopher J Scudder, Samantha M Mirczuk, Karen M Richardson, Victoria J Crossley, Jacob T C Regan, Ruth Gostelow, Yaiza Forcada, Katarina Hazuchova, Norelene Harrington, Imelda M McGonnell, David B Church, Patrick J Kenny, Márta Korbonits, Robert C Fowkes, Stijn J M Niessen

**Affiliations:** 1 Diabetic Remission Clinic, Department of Clinical Science and Services, Royal Veterinary College, North Mymms, United Kingdom; 2 Endocrine Signaling Group, Department of Comparative Biomedical Sciences, Royal Veterinary College, London, United Kingdom; 3 Pathobiology and Population Sciences, Royal Veterinary College, London, United Kingdom; 4 Comparative Biomedical Sciences, Royal Veterinary College, London, United Kingdom; 5 SASH Vets, Neurology and Neurosurgery, Sydney, New South Wales, Australia; 6 Department of Endocrinology, William Harvey Research Institute, Barts and The London School of Medicine, Queen Mary University of London, London, United Kingdom; 7 The Diabetes Research Group, Institute of Cellular Medicine, University of Newcastle, Newcastle, Tyne and Wear, United Kingdom

**Keywords:** acromegaly, hypersomatotropism, somatostatin, dopamine receptor, cat

## Abstract

The prevalence of GH-secreting pituitary tumors in domestic cats (*Felis catus*) is 10-fold greater than in humans. The predominant inhibitory receptors of GH-secreting pituitary tumors are somatostatin receptors (SSTRs) and D_2_ dopamine receptor (DRD2). The expression of these receptors is associated with the response to somatostatin analog and dopamine agonist treatment in human patients with acromegaly. The aim of this study was to describe pathological features of pituitaries from domestic cats with acromegaly, pituitary receptor expression, and investigate correlates with clinical data, including pituitary volume, time since diagnosis of diabetes, insulin requirement, and serum IGF1 concentration. Loss of reticulin structure was identified in 15 of 21 pituitaries, of which 10 of 15 exhibited acinar hyperplasia. *SSTR1*, *SSTR2*, *SSTR5*, and *DRD2* mRNA were identified in the feline pituitary whereas *SSTR3* and *SSTR4* were not. Expression of *SSTR1*, *SSTR2*, and *SSTR5* was greater in acromegalic cats compared with controls. A negative correlation was identified between *DRD2* mRNA expression and pituitary volume. The loss of DRD2 expression should be investigated as a mechanism allowing the development of larger pituitary tumors.

Acromegaly is typically caused by a functional GH-secreting pituitary adenoma in humans, and this results in increased circulating IGF1 [1]. Medical management therapies for acromegaly include GH receptor antagonists, dopamine receptor agonists, and somatostatin analogs, with the latter being the medical therapy of choice in most cases [2, 3]. However, 30% to 65% of patients with acromegaly receiving somatostatin analogs for 12 months fail to achieve biochemical disease control [4–6]. This limited response to therapy is justification for ongoing research to develop therapies that improve outcomes in medically managed patients [7].

Animal models can provide insight into disease pathophysiology and are used for therapeutic drug development. Transgenic rats, mice, and rabbits are commonly used as induced acromegalic models by overexpression of GHRH or aryl hydrocarbon receptor–interacting protein knockout [8–11]. However, these models do not replicate GH-secreting pituitary adenomas identified in most human patients with acromegaly, and this might limit the predictability of pharmacological studies of tumorous pituitary GH-secretion inhibition when using them. Additionally, the study of a naturally occurring disease from an animal that lives in a similar environment to humans would be favorable to account for the potential environmental effects on pituitary dysfunction.

Spontaneous acromegaly/hypersomatotropism (HST) in domestic cats (*Felis catus*) is 10-fold more prevalent than in humans, affecting an estimated 1 in 800 cats [12–14]. Acromegaly in cats parallels the disease in humans as far as being diagnosed in middle-aged to older subjects and is associated with insulin resistance, acral growth, and cardiovascular complications [12, 15]. Cats affected by acromegaly have achieved long-term clinical and biochemical response to pasireotide and cabergoline but no other medical therapies [16–19]. The somatostatin and dopamine receptor profile of feline GH-secreting adenomas is not known. The receptor expression profile of these tumors might explain the poor response of feline acromegalics to octreotide, which has high binding affinity for, and preferentially binds to, somatostatin receptor (SSTR2), and l-deprenyl, a monoamine oxidase B inhibitor that prolongs the activity of dopamine, but a favorable response to pasireotide treatment [16, 20, 21].

The aim of the study was to investigate whether cats with naturally occurring acromegaly are a suitable model for the human disease, as well as a species of interest from a veterinary perspective. The study sought to describe the pituitary pathological findings, hormone, somatostatin and dopamine receptor expression of cats with and without acromegaly. Additionally, the receptor expression data were compared with clinical data.

## 1. Materials and Methods

The study was approved by the Royal Veterinary College (RVC) Ethics and Welfare Committee (URN 2014 1306).

### A. Animals

Written informed consent was obtained from owners of all enrolled cats. Cats had a diagnosis of acromegaly on the basis of appropriate clinical history, serum IGF1 concentration >1000 ng/mL (reference interval, 200 to 700 ng/mL), which has a 95% positive predictive value for acromegaly [12], and pituitary enlargement diagnosed using intracranial imaging (contrast enhanced CT) or postmortem examination [12]. All acromegalic cats had concurrent diabetes mellitus that was likely to be secondary to acromegaly, and they were receiving lente insulin (Caninsulin; MSD Animal Health, Kenilworth, NJ), protamine zinc insulin (ProZinc; Boehringer Ingelheim, Ingelheim am Rhein, Germany), or glargine insulin (Lantus; Sanofi, Paris, France) (HST group). Nonacromegalic cats who did not have a clinical history consistent with acromegaly or pituitary enlargement, but had undergone postmortem examination and whose owners consented to be enrolled in the study, were consecutively recruited. All cats had previously been patients of the Queen Mother Hospital for Animals (RVC), Beaumont Animals’ Hospital (RVC), or People’s Dispensary for Sick Animals in London, United Kingdom. All cats had been neutered, which is common in the United Kingdom for patient health and population control.

### B. Cat Pituitary Tissue

Pituitary tissue was obtained at the time of postmortem examination or therapeutic hypophysectomy. Tissue was fixed in RNAlater^TM^ (Sigma-Aldrich, Dorset, United Kingdom) or snap frozen in liquid nitrogen and stored at −80°C until processed in batches. A section of pituitary tissue was also fixed in 10% w/v neutral buffered formalin, dehydrated in decreasing concentrations of ethanol, and then embedded into paraffin blocks and stored at room temperature (RT). A summary of clinical characteristics of the enrolled cats is presented in Table 1.

**Table 1.** Clinical Data of Cats in the Control and Acromegalic Groups

	Age (y)	Sex	Body Weight (kg)	Breed	Concurrent Disease	Treatment	Insulin (U/12 h)	Time Diabetic (m)	Pituitary DV Height (mm)	Pituitary Volume (cm^3^)	IGF1 (ng/mL)
Control group											
1	11	M	3.7	Tonkinese	DM	Insulin: lente	2	5			
2	12	M	5.0	ASH	DM	Insulin: PZI	1.5	16			173
3	14	M	4.7	DSH	DM	Insulin: PZI	2.5	12			468
4	10	M	4.4	DSH	DM	Insulin: lente	4.5	1			
5	15	F	3.3	DSH	DM	Insulin: glargine	1	4			222
6	13	M	5.4	DSH	Cardiomyopathy	Furosemide, pimobendan, clopidogrel					
7	13	F	3.1	DSH	Lymphoma	Prednisolone, vincristine					
8	15	F	3.4	DLH	CKD						
9	1	M	4.6	DSH	CKD	Aluminum hydroxide					
10	6	M	4.3	Oriental	IMHA	prednisolone					
11	2	F	4.6	Savannah	Cardiomyopathy	None					
12	9	M	6.5	Norwegian Forest	Sepsis	Multiple antibiotic therapy					
13	7	M		DSH	Pleural effusion						
14	15	M	4.7	DSH	CKD						
15	16	M	6.6	DSH	DM				4		868
16	8	M	4.0	DSH	DM	Newly diagnosed					
17	2	F	3.5	DSH	Myelodysplasia	Prednisolone, chlorambucil					
18	16	F	3.1	DSH	DM/ hyperaldosteronism	Insulin: glargine, spironolactone		7			
19	12	F	5.2	DSH	CKD						
20	1	F	4.1	DSH	IMHA	Prednisolone, chlorambucil					
21	15	M	4.3	DLH	CKD	Aluminum hydroxide					
22	18	M	3.9	DSH	Gastrointestinal disease: unclassified						
HST group											
1	11	F	6.6	DSH	DM, chronic enteropathy: unclassified	Insulin: lente, PAS-LAR	2	15	6.2	0.15	1598
2	11	M	5.7	DSH	DM	Insulin: glargine, PAS-LAR	2	10	5.0	0.09	>2000
3	10	M	4.9	DSH	DM, hepatopathy: unclassified	Insulin: glargine	0.5	15	10.0	0.58	1271
4	13	M	4.2	DLH	DM	Insulin: lente	7	13	6.6	0.08	1824
5	14	M	4.1	DSH	DM/HCM	Insulin: lente, PAS-LAR, aspirin	3	53	6.4	0.05	1716
6	10	M	8.0	DSH	DM	Insulin: glargine	0.5	4	5.0	N/A	1629
7	13	M	6.5	DSH	DM	Insulin: lente	11	45	7.1	0.13	1885
8	5	M	7.1	DSH	DM	Insulin: glargine	18	24	5.7	0.09	>2000
9	10	M	6.0	DSH	DM	Insulin: lente	11	9	7.0	0.14	>2000
10	6	M	5.0	DLH	DM	Insulin: glargine	3	5	7.8	0.27	1391
11	15	M	5.0	DSH	DM	Insulin: glargine	4	5	7.0	0.12	1536
12	14	M	5.4	DSH	DM, chronic enteropathy	Insulin: glargine	1.5	0	5.8	0.06	1342
13	11	M	5.2	DSH	DM	Insulin: glargine		0	5.5	0.09	>2000
14	6	M	7.2	DSH	DM	Insulin: lente		0	4.5	0.07	1289
15	14	M	4.5	Maine Coon	DM	Insulin: PZI	19	4	6.6	0.08	1847
16	9	M	4.1	DSH	DM	Insulin: lente	5.5	6	6.1	0.09	1322
17	14	M	3.5	DSH	DM, CKD	Insulin: lente	7.5	2	0.0	N/A	1395
18	12	M	5.9	Maine Coon	DM	Insulin: lente	6	4	5.8	0.11	1672
19	10	M	5.6	DSH	DM	Insulin: lente	14	3	9	N/A	1500
20	6	M	3.5	DSH	DM	Insulin: glargine	1	1	5.4	0.07	1287
21	9	M	5.8	DSH	DM	Insulin: lente	2	3	5.5	N/A	>2000
22	8	M	4.3	Maine Coon	DM	Insulin: lente, PAS-SAR	21	5	11.1	0.65	>2000
23	11	F	5.5	DSH	DM, chronic enteropathy	Insulin: glargine, PAS-LAR	15	19	8.5	0.40	>2000
24	8	M	4.6	DSH	DM	Insulin: lente	18	7	11.0	0.61	>2000
25	14	M	5.4	DSH	DM, chronic enteropathy	Insulin: lente	0	5	5.0	0.06	1382
26	10	M	5.4	DSH	DM	Insulin: glargine	3.5	3	5.0	0.05	1567
27	15	M	11.3	DSH	DM	Insulin: lente	0	21	10.0	N/A	1770
28	15	M	4.0	BSH	DM	Insulin: lente	0	8	5.2	0.03	>2000
29	13	M	5.7	DSH	DM, CKD	Insulin: glargine, PAS-LAR	3	21	5.6	0.08	>2000
30	13	F	7.7	DSH	DM	Insulin: lente	4	5	6.3	N/A	1304
31	11	F	5.7	DSH	DM	Insulin: glargine	9	4	7.4	0.17	919
32	7	F	8.0	DLH	DM	Insulin: lente	7	4	6.2	0.12	1875
33	10	M	5.9	Bengal	DM	Insulin: lente	5	3	4.8	0.08	1188
34	9	F	6.6	BSH	DM, chronic enteropathy	Insulin: lente, SAMe	5	5	7.2	0.17	1775
35	12	M	6.7	DSH	DM	Insulin: lente	11	N/R	7.0	0.08	>2000
36	9	M	4.4	DSH	DM	Insulin: lente	14	6	6.8	0.16	>2000
37	14	F	4.8	DSH	DM	Insulin: PZI, PAS-LAR	0.5	13	6.0	N/A	1938
38	13	F	3.5	DSH	DM, hypertrophic cardiomyopathy	Insulin: lente	4	4	5.2	0.06	>2000
39	11	F	3.5	BSH	DM	Insulin: PZI	9	24	5.4	N/A	1210

All cats enrolled in this study were neutered.

Abbreviations: ASH, American shorthair; BSH, British shorthair; CKD, chronic kidney disease; DLH, domestic longhair; DM, diabetes mellitus; DSH, domestic shorthair; F, female; HCM, hypertrophic cardiomyopathy; IMHA, immune-mediated hemolytic anemia; M, male; N/A, not applicable; N/R, not recorded; PAS-LAR, pasireotide long-acting release; PAS-SAR, pasireotide short-acting release; PZI, protamine zinc insulin; SAMe, *S*-adenosyl-l-methionine.

### C. Reticulin Staining

Tissue sections were cut, deparaffinized and rehydrated as follows: 4-µm sections were cut using a manual rotary microtome (Leica RM2235; Leica Biosystems, Newcastle upon Tyne, United Kingdom) and air dried onto microscope slides (Superfrost^TM^ microscope slides; Thermo Fisher Scientific, Loughborough, United Kingdom); deparaffinization of the sections was performed by heating slides to 60°C for 5 minutes followed by two 5-minute immersions in HistoClear (National Diagnostics, Atlanta, GA) or xylene (Sigma-Aldrich) and rehydration of tissues in decreasing concentrations of ethanol. A commercially available reticulin staining kit (reticulin stain ab150684; Abcam, Cambridge, United Kingdom) was used, and the procedure was performed as per the manufacturer’s guidelines apart from use of 1 M ammonium hydroxide, where the kit describes use of “concentrated ammonium hydroxide” to make the “working ammoniacal silver solution.” A feline kidney tissue section was used as a positive control for each batch of reticulin fiber staining.

Ten control pituitary samples were used to develop a reference interval for the number of nuclei within each acinus and area of each acinus. Ten acini from each sample were randomly selected from each pituitary. This resulted in 100 acini being used for reference interval determination. This reference interval was then tested using two other control pituitary samples. Three assessors (Dr. Christopher Scudder, Katarina Hazuchova, Veterinary Internal Medicine Specialist, and Norelene Harrington, Specialist in Veterinary Pathology) were used to determine whether pituitary acinar morphology was altered in pituitaries from cats with acromegaly. Each assessor was asked the following questions: Is the acinar structure altered? Are the acini increased in size? Is there loss of acinus structure? Is the distribution focal, multifocal, or diffuse? Loss of acinus structure would be consistent with adenomatous change, and an increased size of acini would be consistent with acinar hyperplasia. The upper reference limit for acinar size is described in “Reticulin Staining” in the “Results,” and the responses to the above questions were used to determine a consensus among assessors.

### D. Immunohistochemistry

All pituitary samples used for immunohistochemistry had previously undergone hematoxylin and eosin staining. Pituitary tissue embedded in paraffin blockswas cut into 4-µm sections and air dried on positively charged slides (Superfrost^TM^ Plus microscope slides; Thermo Fisher Scientific, Loughborough, United Kingdom). Immunohistochemistry was performed as previously described [22] by deparaffinization and rehydration of the sections as per reticulin staining. Antigen retrieval for GH immunostaining was not necessary. Antigen retrieval for prolactin (PRL) and SSTR2 quantification was required. For PRL immunostaining, slides were immersed in a pH 9.0 Tris-EDTA buffer (10 mM Tris base, 1 mM EDTA solution, 0.05% Tween^®^ 20), followed by microwave heating at 650 W for 4 minutes four times. For SSTR2 immunostaining, slides were immersed in 10 mM citrate buffer (pH 6) and microwave heating at 650 W for 4 minutes four times. Slides were cooled to RT for 30 minutes followed by blocking of endogenous peroxidase by immersion in 3% v/v H_2_O_2_ for 10 minutes. Nonspecific protein binding was blocked by immersion in a buffer containing PBS (Gibco, Thermo Fisher Scientific, Loughborough, United Kingdom), 5% goat serum (Sigma-Aldrich), 1% BSA (Sigma-Aldrich), 0.1% w/v Triton™ X-100 (Sigma-Aldrich), and 0.05% Tween^®^ 20 (Thermo Fisher Scientific, Loughborough, United Kingdom).

Primary antibody incubation was performed overnight in a cold room. Rabbit anti-porcine GH and rabbit anti-porcine PRL antibodies were used [23, 24]. The primary antibodies were delivered lyophilized and reconstituted using PBS to a concentration of 1 mg/mL for anti-porcine GH antibody and 300 µg/mL for anti-porcine PRL antibody as per the manufacturer’s guidelines. Primary antibody incubation used anti-porcine GH at 1:6000 dilution, anti-porcine PRL at 1:4000 dilution, and anti-SSTR2 [25] at 1:1600 dilution. Secondary antibody incubation was performed using species-specific biotinylated antibodies (Vector Laboratories, Peterborough, United Kingdom) for 30 minutes at RT followed by incubation with avidin/biotin complex (Vector Laboratories) for 30 minutes at RT. Slides were then incubated with diaminobenzidine (DAB) chromogen (Vector Laboratories) for 2 minutes, followed by counterstaining using Gills hematoxylin for 40 seconds at RT. Between each step the slides were washed in PBS and 0.05% Tween^®^ 20 for 5 minutes three times. Tissues were dehydrated in increasing concentrations of ethanol and then slides were coverslipped using Vectashield antifade mounting medium (Vector Laboratories) and analyzed. Negative control samples underwent immunohistochemistry as described above but without addition of the primary antibody, and positive control samples were sections from a healthy mouse pituitary for GH and PRL, and from a healthy human pituitary for SSTR2 immunostaining.

**Figure 1.**
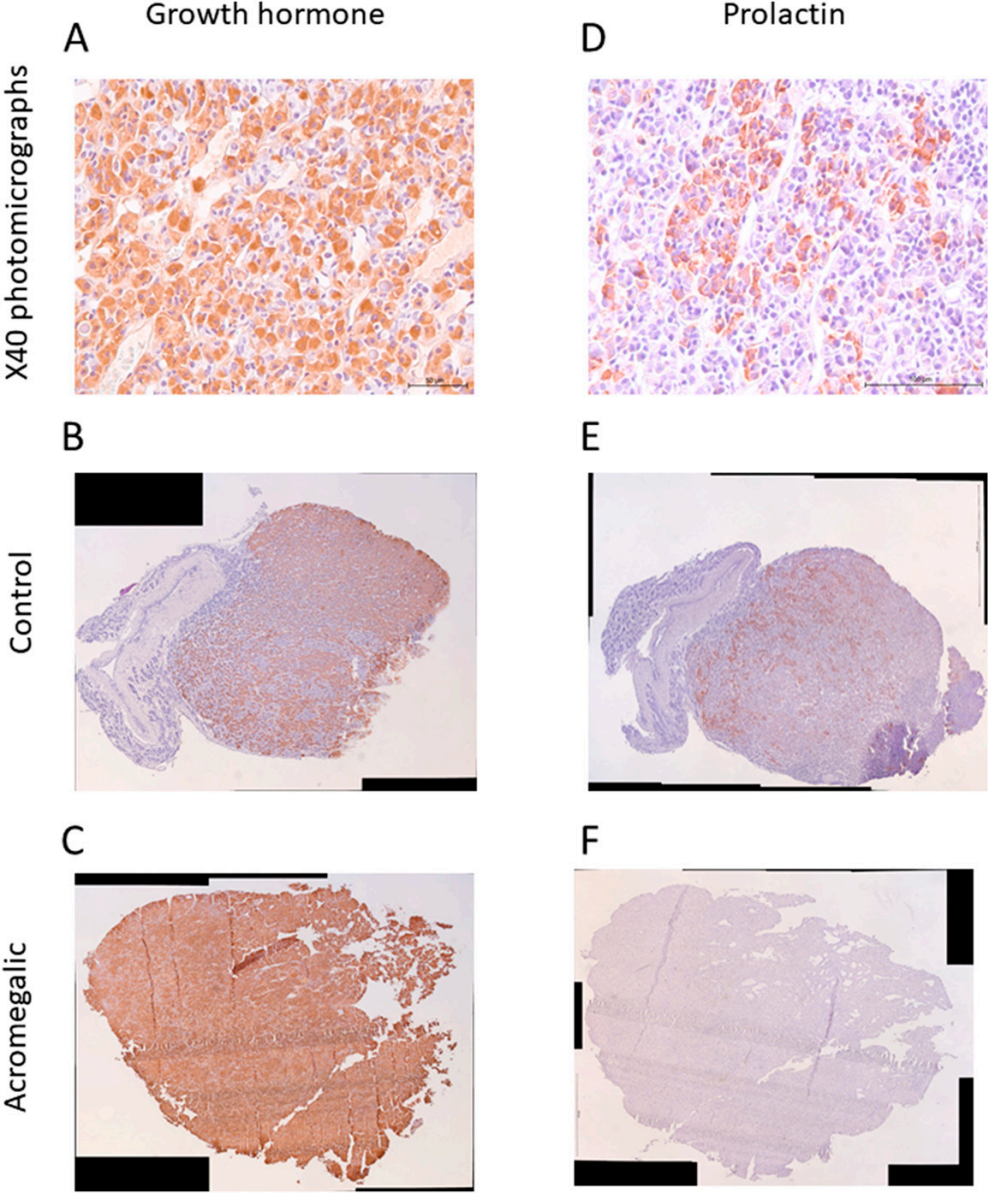
Representative photomicrographs of GH (A–C) and PRL (D–F) immunostaining. (A and D) Immunoreactivity is identified by diaminobenzidine (DAB) chromogen and tissue counterstaining performed using Gill hematoxylin. Photomicrographs demonstrating specific immunostaining for somatrophs and lactotrophs, respectively. Original magnification, ×40 (B and E). Photomicrographs of B–C and E–F were created by obtaining high-resolution photomicrographs at magnification ×100 and stitching images from each tissue together using image editing software. (B and E) Photomicrographs from a control cat. (C and F) Photomicrographs from an acromegalic cat.

**Figure 2.**
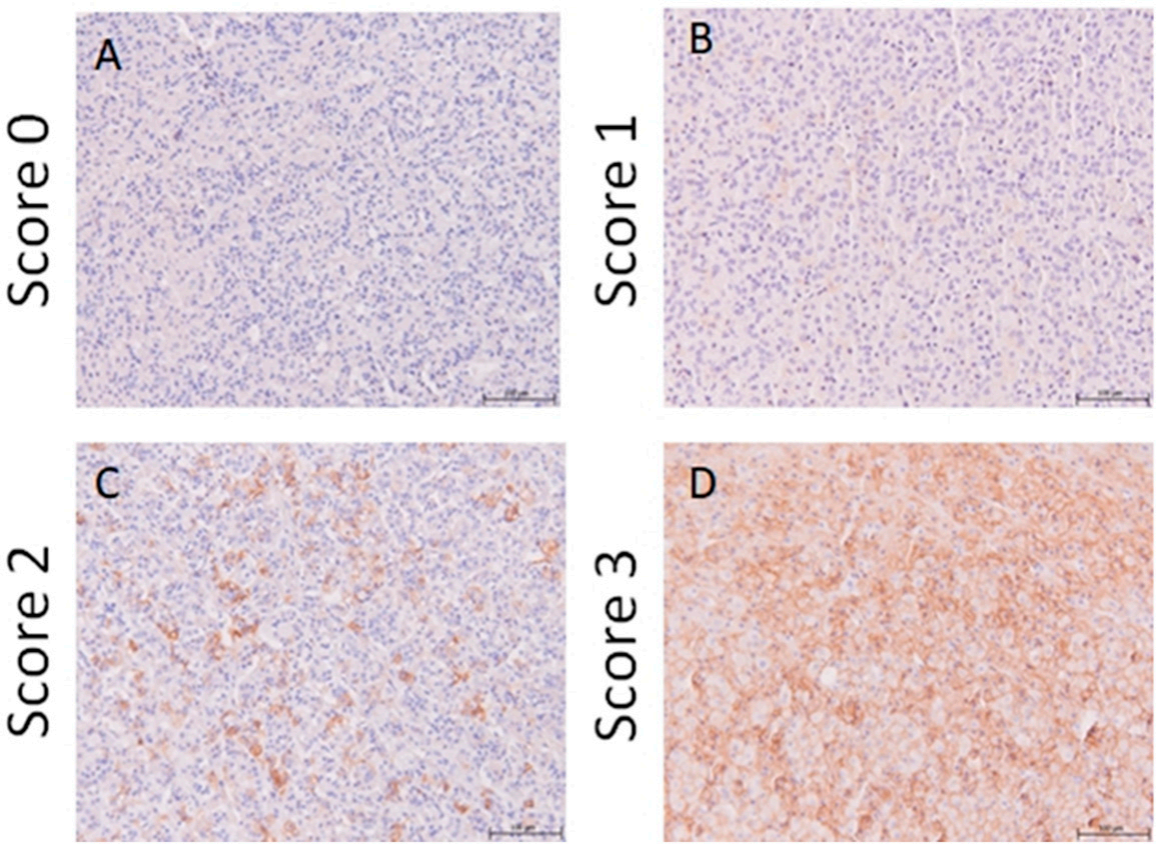
Representative images of SSTR2 immunoreactivity using feline pituitary tissue. (A–D) Immunoreactivity is identified by diaminobenzidine (DAB) chromogen and tissue counterstaining performed using Gill hematoxylin. Pituitary tissue exhibiting SSTR2 immunohistochemistry scores 0, 1, 2 and 3, respectively, using the following criteria: 0, absent; 1, cytoplasmic staining; 2, membranous staining in <50% cells or incomplete membranous staining; and 3, circumferential membranous staining in >50% cells. All presented photomicrographs were collected at an original magnification of ×100.

Representative immunostaining for GH and PRL are presented in Fig. 1. The percentage DAB immunoreactivity of each tissue section was determined by obtaining high-resolution photomicrographs at magnification ×100 (Leica DM4000 B; Leica Microsystems, Milton Keynes, United Kingdom) and stitching images from each tissue together using image editing software (Microsoft Image Composite Editor 2.0 for Windows; Microsoft, Redmond, WA) to create a digital copy of the tissue. Area measurements were performed using Volocity version 6.3.0 (PerkinElmer, Waltham, MA). The area of DAB labeling was detected by thresholding of hue and saturation. Any contiguous object <5 pixels was considered noise and excluded before the total area of the detected object was calculated. The total tissue area was also detected and used to calculate percentage DAB positivity of each tissue. Scoring of sections that used anti-SSTR2 antibodies as the primary antibody was also performed by three individuals in a blinded manner using a semiquantitative scale as previously described [26]. Immunoreactivity intensity was graded 0 to 3 (0, absent; 1, cytoplasmic staining; 2, membranous staining in <50% cells or incomplete membranous staining; and 3, circumferential membranous staining in >5% cells; see Fig. 2 for examples). When there was a conflict of the pituitary score between one reviewer but two agreed then the agreed-upon score was used, and when all three reviewers disagreed then the average score was used.

### E. Pituitary RNA Extraction, Analysis, and Selection of Reference Genes

Pituitary RNA was extracted from 10 cats without pituitary disease using the phenol chloroform technique. The RNA pellet was resuspended in RNase-free water and underwent on-column DNase treatment using a commercially available kit and following the manufacturer’s instructions (RNeasy Maxi kit; Qiagen, Manchester, United Kingdom). RNA quantity and integrity were assessed using the Nanodrop™ 1000 spectrophotometer (Thermo Fisher Scientific, Hemel Hempstead, United Kingdom) and Agilent 2100 Bioanalyzer (Agilent Biotechnologies, Craven Arms, United Kingdom).

An aliquot of 100 ng of total pituitary RNA was used to synthesize first-strand cDNA using 1 µL of oligo(dT) primer (Promega, Madison, WI) and ImProm-II^TM^ reverse transcription system (Promega) per the manufacturer’s instructions with added magnesium chloride (50 mM MgCl_2_; Bioline London, United Kingdom). The cDNA was eluted using 100 µL of RNase-free water and stored at −20°C until batch use. A nonreverse-transcribed sample was prepared as a control for each sample. The selection of the reference genes for GeXP multiplex was performed using the geNorm algorithm [27] and feline geNorm 6 gene kit for use with SYBR Green (PrimerDesign, Southampton, United Kingdom). An *m* value of <0.5 was the cutoff for selection. *RPL18* and *SDHA* were chosen as the reference genes.

### F. Multiplex RT-qPCR

**Figure 3.**
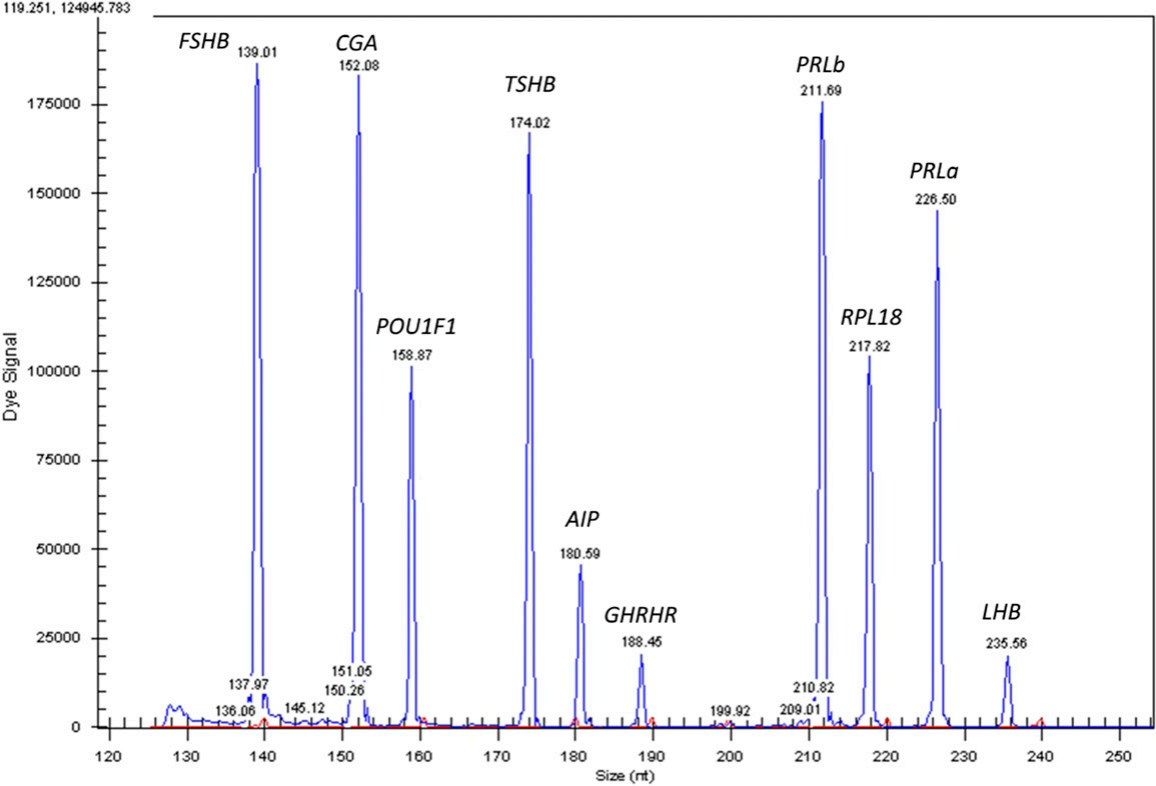
Electropherogram results from PCR products using multiplex 1 primer sets. The blue peaks represent PCR products from gene-specific primers and the red peaks represent product size standards.

Three custom-designed GeXP multiplexes (GenomeLab gene expression profiler; Beckman Coulter, Wycombe, United Kingdom) were used to quantify gene expression. Multiplex 1 consisted of primers designed for *AIP*, *CGA*, *FSHβ*, *GHRHR*, *LHβ*, *PRL*, *POU1F1*, *TSHβ*, *RPL18*, and *SDHA*, multiplex 2 consisted of primers designed for *POMC*, *GH1*, *RPL18*, and *SDHA*, and multiplex 3 consisted of primers for *SSTR1*, *SSTR2*, *SSTR3*, *SSTR4*, *SSTR5*, *DRD2*, *RPL18*, and *SDHA* [28]. There were two primer sets for the measurement of *PRL*, labeled as PRLa and PRLb, to investigate the precision of gene amplification using the GeXP technique. The GeXP multiplex was performed as previously described and in accordance with the manufacturer’s instructions [29, 30]. This procedure uses the GeXP start-up kit (Beckman Coulter) to synthesize cDNA using gene-specific antisense primers with a 3′ universal tag reverse sequence and 100 ng of total pituitary RNA using a G-Storm GS1 thermal cycler and the following protocol: 48°C, 1 minute; 42°C, 60 minutes; and 95°C, 5 minutes. Following first-strand cDNA synthesis, an aliquot from each reaction was added to a PCR master mix containing GenomeLab kit PCR master mix and DNA polymerase (Thermo-Start DNA polymerase; Thermo Fisher Scientific, Loughborough, United Kingdom). PCR reaction was performed using G-Storm GS1 thermal cycler and the following protocol: 95°C for 10 minutes, followed by 35 cycles at 94°C for 30 seconds, 55°C for 30 seconds for multiplex 1 and 3 and 65°C for multiplex 2, and 70°C for 60 seconds. Products were analyzed by separation using capillary electrophoresis followed by fluorescence spectrophotometry and quantified using CEQ^TM^ 8000 genetic analysis system and GenomeLab fragment analysis software (Beckman Coulter). Examples of electropherograms for multiplex 1 and 3 are presented in Fig. 3. Because many samples had SDHA and POMC expression below the level of detection, RPL18 was used as the sole reference gene and the difference between groups of POMC expression was not undertaken.

### G. Statistical Analysis

Data were visually assessed for normal distribution using histograms and by performing Shapiro–Wilk tests. Normally distributed data are described as mean and SD and nonnormally distributed data as median and interquartile range (IQR). Statistical significance was determined using an unpaired *t* test and Mann–Whitney test. A Spearman rank correlation or Pearson correlation was used to test the association between gene expression and clinical variables. Agreement of SSTR2 scores between observers was assessed using a two-way random effects single measures intraclass correlation coefficient for absolute agreement model. A *χ*^2^ test was used to test the SSTR2 scores between acromegalic and control groups. A *P* value of <0.05 was considered significant, and a Holm-Bonferroni adjustment was used for adjustment of multiple comparisons where appropriate. Statistical software analyses were performed using GraphPad Prism version 7.02 for Windows (GraphPad Software, La Jolla, CA) and IBM SPSS Statistics for Windows version 22 (IBM Corporation, New York, NY).

## 2. Results

### A. Reticulin Staining

**Figure 4.**
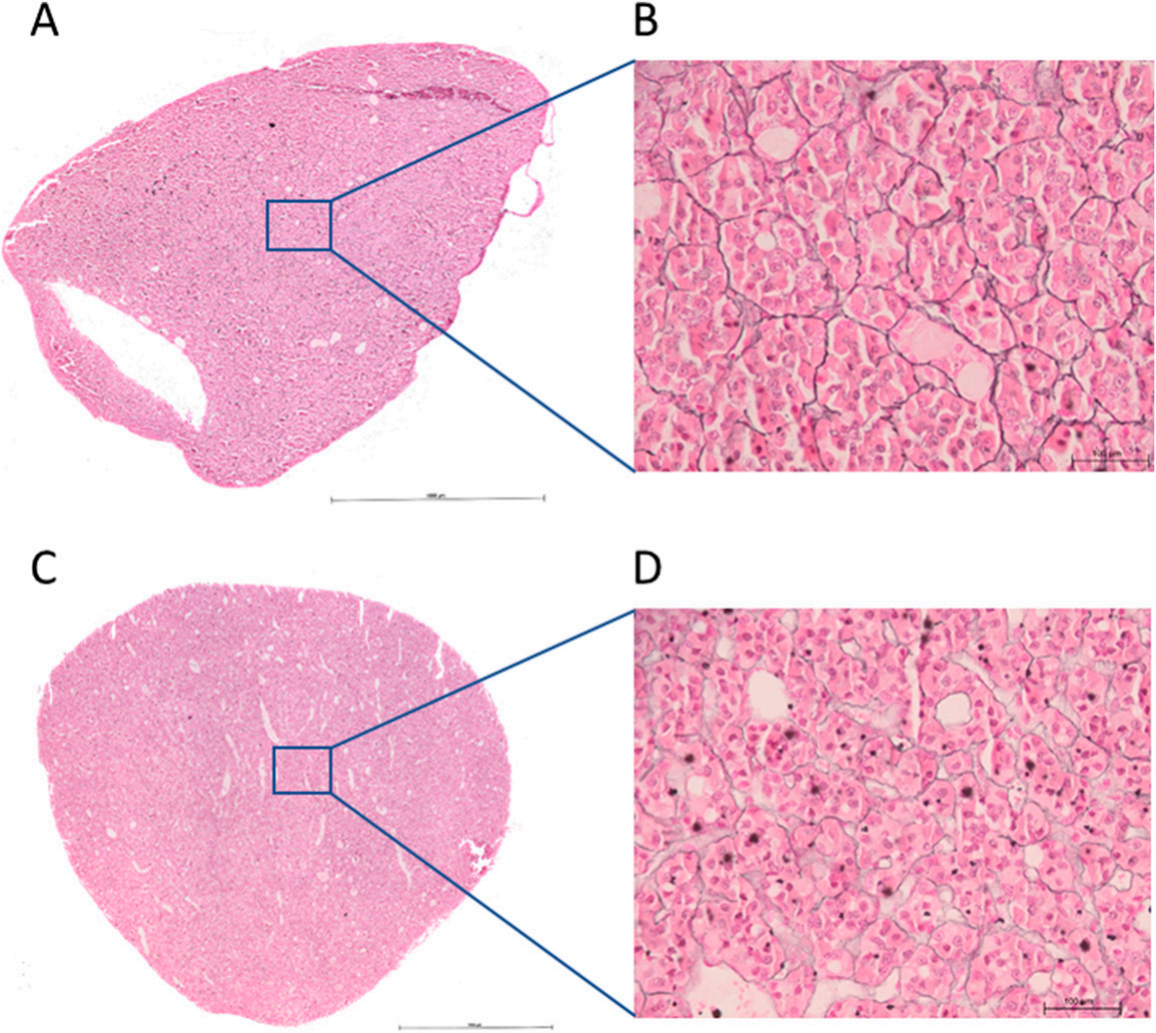
Images stained using silver stain for reticulin fibers and counterstained using nuclear fast red solution. (A and C) Reconstructed stitched pituitary photomicrographs from two control pituitaries. Original magnification, ×100. (B and D) Enlarged photomicrographs from sections of (A) and (C), respectively. Original magnification, ×400. The acinar pattern of reticulin staining is identified in (B) and (D). This pattern of reticulin staining was demonstrated in all reticulin staining control pituitaries.

**Figure 5.**
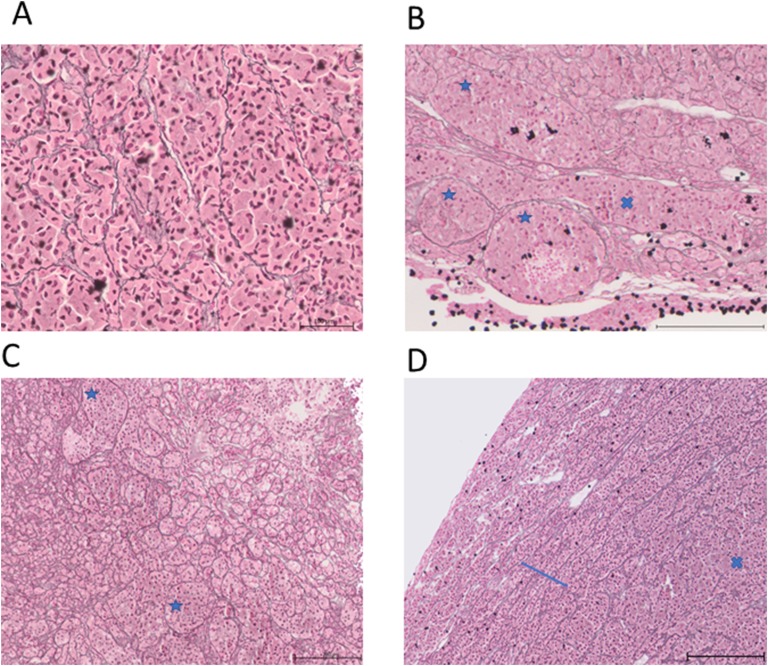
All images stained using silver stain for reticulin fibers and counterstained using nuclear fast red solution. (A–D) Selected images taken from reconstructed stitched pituitary photomicrographs from four HST pituitaries. Original magnification, ×100. (A) Disrupted reticulin staining and loss of acinar structure. (B) Areas of enlarged acini (blue stars) and areas of loss of acinar structure (blue cross). (C) Enlarged acini (blue stars) adjacent to normal size and small acini. (D) Loss of acinar structure in the bottom right of the image (blue star); adenomatous tissue has compressed the normal pituitary tissue, resulting in compression of the acini and a ring of cords of acini giving the impression of a pseudocapsule.

The reticulin staining in the control pituitary glands demonstrated an acinar and cords pattern (Fig. 4). This pattern is the same as described in the healthy human pituitary gland [31]. The upper reference interval for the number of nuclei per acinus in the control pituitary samples was 66, and the upper reference interval for the area of each acinus was 12,650 µm^2^. The two remaining control pituitary samples were assessed using this scoring system and both were considered within normal limits. A spectrum of altered reticulin staining was identified in the HST pituitary samples, including enlargement of acini, disrupted reticulin staining, and loss of reticulin staining (Fig. 5). Compression of the normal pituitary parenchyma adjacent to neoplastic tissue was also identified that created a ring of cords of reticulin staining in some tissue samples. Three assessors reported 7 of 21 pituitaries exhibited loss of acinus structure, which was described as diffuse or multifocal in all cases. Of the remaining pituitaries, two assessors (Dr. Christopher Scudder and Katarina Hazuchova for all 8 cases) described a loss of acinus structure in 8 of 14 cases, which was focal in 4 of 8 cases and multifocal or diffuse in the remaining 4 cases. All three assessors described an increased in size of acini in 5of 21 pituitaries. Of the remaining pituitaries, two assessors (Katarina Hazuchova and Norelene Harrington for all 5 pituitaries) described 5 of 16 pituitaries as having enlarged acini. There were no distinguishing clinical features of the 10 cats who were described to exhibit pituitary acinar enlargement (acromegaly cat nos. 7, 14, 22, 24, 25, 32, 34, 35, 36, and 38).

### B. GH and PRL Expression

There was no difference of patient sex (*χ*^2^ test, *P* = 0.334) or patient age (median control vs HST was 11 vs 11 years, Mann–Whitney *U* test, *P* = 0.870) between groups, but there was a difference in body weight (median control vs HST was 4.3 vs 5.4kg, Mann–Whitney *U* test, *P* = 0.006). The difference in body weight between groups was expected and likely due to the acromegalic state.

There was significantly greater GH protein expression in the HST compared with control group (mean, 50% ± 27% vs 30% ± 21%; *t*(51) = 2.914, *P* = 0.005; Table 2). Although gene expression of *GH1* was greater in cats with acromegaly than in controls, this was not statistically significant (median control vs HST was 3.1 vs 6.2; Mann–Whitney *U* test, *P* = 0.071). There was no difference of PRL protein or gene expression between the HST and control group [median protein expression, 1.5% (IQR, 10.9) vs 4.1% (IQR, 4.2); Mann–Whitney *U* test, *P* = 0.122; median relative gene expression, 2.099 (IQR, 1.7) vs 2.196 (IQR, 0.73); Mann–Whitney *U* test, *P* = 0.033]. There was no correlation between patient age and GH or PRL expression, nor was there an association between age and any pituitary gene expression in this study.

**Table 2.** Gene Expression Data and GH, PRL, and SSTR2 Immunohistochemistry Scoring of Cats in the Control and Acromegalic Groups

	Relative Gene Expression																IHC % DAB+		IHC % DAB+: *SSTR2*	Reticulin Staining
	*CGA*	*FSHB*	*GH1*	*LHB*	*PRL*	*TSHB*	*SSTR1*	*SSTR2*	*SSTR5*	*DRD2*	*AIP*	*GHRHR*	*GHSR*	*ESR1*	*ESR2*	*GPER1*	*GH*	*PRL*		
Control group																				
1	4.235	4.868	3.252	0.175	2.324	3.114	0	0	0.0123	0.643	0.3728	0.1996	0.0603	0.2047	0.4541	0.6316				
2	5.500	8.317	3.666	0.307	1.502	2.709	0.01	0	0.0606	1.3891	0.4833	0.236	0.1165	0.4354	0.504	2.2935				
3		16.915	1.553	1.178	1.436	1.886	0.01	0	0.0321	1.3724	2.7279	0.2031	0.0886	0.1574	0.5736	0.5146				
4	2.341	2.536		0.080	1.399	0.844					0.2895	0.3056	0	0.0621	0.3556	0.4907	1.704	3.728	0.346	
5	5.802	4.775	1.173	0.546	2.918	2.524					0	0	0	0.2755	0.8168	1.9429				
6	4.453	4.742		0.506	2.099	0.952	0.03	0	0.0218	1.3121	0.2923	0.322	0.2332	0.2005	0.7558	1.6494	75.246	4.036	0.000	x
7	17.060	20.215			7.620	13.866	0.01	0.0027	0.0224	1.0812	0.3575	0.2141	0.1753	0.3414	0.9114	0.5207	62.875	4.194	0.864	x
8	5.708	5.612	3.111	0.226	3.405	3.550	0	0	0	1.1168	0.5914	0	0	0	0.0964	0.5344	29.303	6.927	0.013	
9	2.937	2.857	1.673	0.443	1.965	2.223	0	0	0.0223	1.6752	0	0	0.2845	0	0.21	0.6574	45.826	1.557	0.057	x
10			3.279														0.525	4.168		
11			5.127				0	0.0132	0.0653	0.9713	0.296	0.3148	0.2885	0	0.3502	0	7.613	8.105		x
12							0.01	0.0063	0.0674	0.9928	0.2788	0.2802	0.286	0	0.4769	0.3031	10.796	0.590	0.001	x
13																	42.158	17.888		x
14																	30.946	2.522	0.020	x
15																	31.253	1.994	0.097	
16																	39.220	0.000		
17																	40.529	12.876	0.003	x
18																	16.014	12.111		x
19																	15.324	0.066	0.003	x
20																	50.075	12.475	0.000	x
21																	41.906	18.296	7.919	x
22																	7.965	0.155	0.020	
HST group																				
1	1.604	2.116		0.031	1.598	0.562	0.11	0.0651	0.2217	0.7414	0.4326	0.2955	0.12	0.1245	0.2187	0.3345				
2	1.612	1.585	3.713	0.050	1.840	1.105	0.11	0.0223	0.3064	0.8585	0.3928	0.4603	0.1759	0.0559	0.4643	0.6155	95.615	1.256	1.269	x
3	1.500	0.574		0.147	0.646	0.097	0	0	0.0368	0.1674	0.4958	0.057	0.0201	0.1177	0.9463	1.4239				
4	2.527	2.585			1.978	2.035	0.25	0	0.2295	0.9474	0.3919	0.2925	0.2299	0.1867	0.6055	0.5994	94.662	0.071	0.020	
5	6.742	7.484	6.750		1.256	1.202	0.03	0	0.0755	1.2558	0.6529	0.3755	0.2365	0.3491	0.8696	0.6269	88.505	0.000	0.008	
6	4.522	5.488		0.325	2.588	2.432	0.01	0.0399	0.0888	1.6544	0.3397	0.2428	0.114	0	0.5608	1.1691				
7	3.088	3.159	2.927	0.199	2.485	0.878	0.26	0.0631	0.3101	1.2568	0.2912	0.4277	0.3374	0	0.354	0.7307	63.758	1.060	2.918	x
8	6.023	6.820		0.096	1.865	3.042	0.03	0.0178	0.1172	0.5834	0.3269	0.2541	0.0757	0.1854	0.3736	0.3201	59.965		0.219	
9	1.805	1.300		0.035	2.115	2.332	0.04	0	0.2361	0.7431	999	999	0.227	0.0101	0.2126	0.5632	55.064	7.518	0.579	x
10	3.367	5.492			2.138	0.996	0.19	0.1519	0.1121	0.8297	0.5192	0.4121	0.2895	0.0301	0.474	0.6729	22.791	5.012	21.549	x
11	4.138	4.230	1.893	0.237	2.537	2.151	0	0.0274	0.0545	2.5064	0.3744	0.1422	0.0206	0.0426	0.3217	0.3696	12.869	20.613	1.091	x
12	3.694	4.490	4.046	0.119	2.402	1.040	0.13	0.0716	0.1226	1.1431	0.2437	0.3611	0.0867	0.3095	0.6685	0.2753	31.142	0.688	0.300	
13	37.371	45.779			11.229	26.894	0.09	0.0064	0.1566	0.7815	0.3222	0.3158	0.2002	0.162	0.5108	0.8293	9.999	0.521		
14	4.058	4.561		0.329	2.906	2.251					1.2654	0.2225	0	0.2448	0.8086	1.2097	39.605	1.661		x
15	5.457	5.663	2.825	0.764	2.254	3.903	0.2	0.0331	0.0916	0.9225	0.3447	0.3162	0.1094				8.009	0.471		
16	5.550	5.751		0.252	3.401	2.319	0.02	0.0576	0.1575	1.0134	0.3192	0.3566	0.1307	0.4555	1.0182	0.7893				
17			7.507				0.01	0.0204	0.0965	1.1995	0	0	0.1035	0	0.5831	0.5592	23.469	0.039	0.018	
18			27.799														83.557	1.992	0.096	x
19			3.442														48.228	1.008	0.022	
20			6.247														56.438	0.857	0.166	
21			15.611														86.845	2.619	0.007	
22			0.938														56.492	6.212	0.342	x
23			6.802														59.583	3.422		
24			15.611														91.761	0.009		x
25			2.207														32.149	2.877	7.421	x
26																	40.870	10.524	0.184	
27																	9.727	8.765	0.167	x
28																	40.353	0.579	0.094	x
29																	34.545	0.359	0.109	x
30																	67.555	0.242		x
31																	94.014	0.000	0.043	x
32																	51.611	1.128	4.888	x
33																	69.541	16.261		x
34																	55.946	3.075		x
35																	82.171	4.575	0.008	x
36																	56.666	0.305	0.016	x
37																	15.673	10.449		
38																	26.292	4.439		x
39																	47.771	4.571		

Abbreviation: IHC, immunohistochemistry.

### C. SSTR2 Expression

There was no difference of patient sex (*χ*^2^ test, *P* = 0.150), age (mean controls vs HST, 10.5 ± 5.9 vs 11 ± 3; *t*(34) = 0.392, *P* = 0.687) but there was a difference in body weight between groups (median control vs HST was 4.1 vs 5.5 kg; *P* = 0.004).

There was agreement between observers for tissue SSTR2 scores (intraclass correlation, 0.57; 95% CI, 0.34 to 0.73; *P* < 0.001). Because of the low number of tissues having scores of 0 and 3, groups 0 to 1 and 2 to 3 were grouped together. There was no difference of proportions of SSTR2 scores between acromegalic and control groups. The tissue percentage DAB positivity results are shown in Table 2. The percentage DAB-positive tissue for SSTR2 immunoreactivity was greater in the HST group than in controls (0.20% vs 0.016%; *P* = 0.026). Nine samples had both *SSTR2* expression data and SSTR2 immunohistochemistry data. A positive correlation between *SSTR2* gene expression and percentage tissue DAB staining was detected (*r*^2^ = 0.76, *P* < 0.001).

### D. Expression of Remaining Anterior Pituitary Hormone and Regulatory Receptor Genes

Five cats with HST had previously received pasireotide treatment. There was no difference of any gene expression data in pasireotide-treated and untreated cats; therefore, pasireotide-treated patients were not excluded. There were no differences between sex or ages of patients between groups for expression data of *CGA*, *GH1*, *FSHβ*, *PRL*, *TSHβ*, *DRD2*, *SSTR1*, *SSTR2*, and *SSTR5*.

Expression of *FSHβ*, *PRL*, and *TSHβ* was detected in all pituitaries (Table 2). Expression of *CGA* was not detected in one control pituitary, and *LHβ* expression was not detected in one control and four HST pituitaries. There were no significant differences of hormone expression between control and HST pituitaries. In the HST group, there were strong correlations of gene expression between the following hormones after adjustment of the *P* value for multiple testing: *CGA* and *FSHβ*, *CGA* and *TSHβ*, and *FSHβ* and *TSHβ*; there was moderate correlation between *PRL* and *TSHβ* (Table 3).

**Table 3.** Summary of Spearman Rank Correlation Gene Expression Data in the Control Group and Acromegalic Groups

Group	Gene	Correlate to	Gene	Spearman Rho	P Value	Adjusted P Value
Control	*PRL*	Versus	*TSHβ*	0.800	0.010	0.104
	*CGA*	Versus	*PRL*	0.810	0.015	0.104
	*CGA*	Versus	*FSHβ*	0.786	0.021	0.104
	*CGA*	Versus	*TSHβ*	0.714	0.047	0.150
Acro	*CGA*	Versus	*FSHβ*	0.979	< 0.001	0.005
	*CGA*	Versus	*TSHβ*	0.937	< 0.001	0.005
	*FSHβ*	Versus	*TSHβ*	0.930	< 0.001	0.005
	*CGA*	Versus	*PRL*	0.615	0.033	0.092
	*FSHβ*	Versus	*PRL*	0.615	0.033	0.092

Abbreviation: Acro, acromegalic.

**Figure 6.**
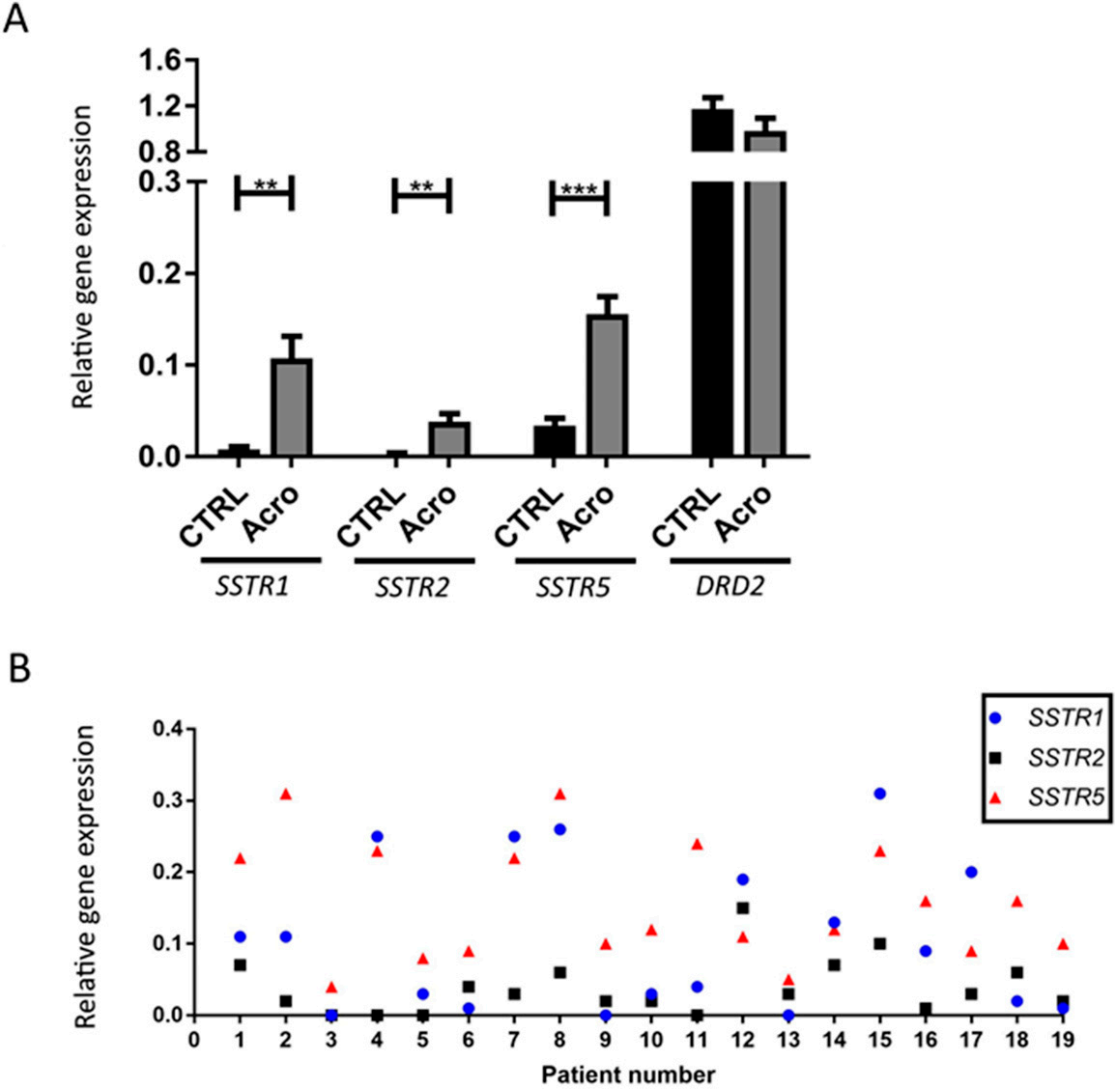
(A) Bar charts comparing the relative gene expression of *SSTR1*, *SSTR2*, and *SSTR5* in pituitary tissue from control (CTRL) and acromegalic (Acro) cats determined using the GeXP multiplex technique. *RPL18* is the reference gene. Bar height represents mean, and error bars are 95% CIs. **P < 0.01, ***P < 0.001. (B) Dot plot of the individual somatostatin profiles from each of the 19 acromegalic cats.

The results of the expression of the *SSTR1*, *SSTR2*, *SSTR5*, and *DRD2* for individuals with HST are shown in Fig. 6. The expression of *SSTR3* or *SSTR4* was not detected. All remaining receptors were detected in 14 of 19 of the HST group, with *SSTR5* and *DRD2* detected in all of the HST group. There was significantly greater expression of SSTR1, SSTR2, and SSTR5 in the HST group compared with controls [0.093 vs 0.008, Mann–Whitney *U* test, *P* = 0.007; 0.036 vs 0.002, *t*(25) = −3.34, *P* < 0.001; 0.151 vs 0.034, Mann–Whitney *U* test, *P* = 0.004] (Fig. 3A). There was highly variable interpatient and intrapatient expression of *SSTR1*, *SSTR2*, and *SSTR5* mRNA in control and HST cats; there was moderate correlation between *SSTR1* and *SSTR5* expression in the HST group (Spearman rho, 0.65; *P* = 0.005); in the control group this correlation was not statically significant (Spearman rho, 0.71; *P* = 0.18). No other receptor expression was correlated with one another. There was a moderate negative correlation between *DRD2* expression and pituitary volume within the HST group (Spearman rho, −0.52; *P* = 0.041). There was no association between somatostatin receptor expression and IGF1 reduction due to pasireotide treatment in the cats that had received pasireotide prior to pituitary tissue collection. There was also no association between somatostatin receptor expression and insulin dose or length of time receiving exogenous insulin therapy.

## 3. Discussion

Human and feline acromegaly share many clinical commonalities, and the disease appears to be increasing in prevalence in both populations. This might in part be due to increased clinical awareness and improved diagnostic tests. This study describes reticulin staining patterns as well as hormone and regulatory receptor expression in the normal and acromegalic feline pituitary for the first time. A description of the normal feline pituitary gland was required because of the paucity of currently available information.

The percentage of GH- and PRL-positive cells in the normal cat pituitary was lower than reported in adult humans (28% vs 45% and 4% vs 15% to 25%, respectively) [32, 33]. As the predominant cell type of acidophils are GH-secreting cells, the distribution of acidophils within a hematoxylin and eosin–stained anterior pituitary section largely reflects the distribution of the GH-producing cells within the feline pituitary gland in health.

There was no consistent pattern of distribution of GH-producing cells in the normal feline pituitary. These cells were seen to cluster or be evenly distributed throughout the anterior pituitary. This pattern differs from the human pituitary where somatotrophs are predominantly located within the lateral wings [33]. PRL-producing cells tended to form clusters of up to 20 cells. This pattern differs from the distribution in humans where they typically occur singularly. However, in concordance with humans, there was no specific location within the gland where the PRL-producing cells were seen [32]

Mixed GH and PRL adenomas or mammosomatotroph adenomas account for up to 30% of cases of acromegaly in humans [34, 35]. PRL-positive cells accounted for <10% of positive cells in 87% of the acromegalic pituitaries, with the remaining samples containing 10.5%, 10.5%, 16%, and 20.5% of PRL-positive cells. Therefore, mixed GH and PRL adenomas/mammosomatotroph adenomas were not a predominant feature of acromegaly in these cats.

The prevalence of pituitary hyperplasia was greater than anticipated. It has been proposed that hyperplastic change can precede adenomatous transformation in human patients, and somatotroph hyperplasia has been shown to result in somatotroph adenoma formation in GHRH-overexpressing mice [10, 36, 37]. Somatotroph hyperplasia is considered a rare cause of acromegaly in humans [38]. The prevalence of pituitary hyperplasia might be greater than suggested by these results if the progression from hyperplasia to adenoma occurs in cats and the hyperplasia stage is missed because many cats are not diagnosed until the onset of diabetes mellitus.

Cats expressed *SSTR1*, *SSTR2*, and *SSTR5* whereas *SSTR3* and *SSTR4* proved undetectable using the employed methodology. Expression of *DRD2* was identified in all feline pituitaries. Cats displayed a similar pituitary SSTR and *DRD2* profile to humans. These data provide therapeutic targets for the management of acromegaly in cats and substantiates the comparative potential of studying the acromegalic cat as a spontaneously occurring model of the human disease [12].

Previous reports of SSTR mRNA expression in GH-secreting pituitary adenomas in humans describe *SSTR5* > *SSTR2* whereas *SSTR1* and *SSTR3* expression can be highly variable and *SSTR4* expression is absent [39–43]. Immunohistochemical reports describe somatotroph receptor expression as either SSTR2 > SSTR5 or SSTR5 > SSTR2 [44–46]. However, these conflicting reports might have occurred owing to a difference in proportion of sparsely vs densely granulated adenomas in the studied groups. These tumor subtypes, which can be differentiated by electromicroscopy or CAM5.2 immunoreactivity pattern, have been documented to have different somatostatin receptor expression profiles [44, 47, 48]. Protein expression of SSTR2 in cats as assessed by immunohistochemistry scoring was lower than that reported in humans [44, 48]. This may be a reason for the previously underwhelming response to octreotide in acromegalic cats, because SSTR2 expression has been positively correlated with octreotide response in humans [18, 42, 49]. Only one cat in the acromegalic group exhibited diffuse strong SSTR2 expression, which suggests that certain individual cats might be suitable candidates to receive octreotide to manage their acromegaly. The lower SSTR2 expression identified in the cats in this study might be because we did not differentiate between sparsely or densely granulated tumors. Finally, the detected positive correlation between *SSTR2* gene expression as measured by GeXP multiplex and protein levels as measured through immunohistochemistry parallels findings from previous studies, further supporting the robustness of this methodology for within-gene expression assessment [48, 50].

There are several different somatostatin receptor immunostaining scoring systems where immunoreactivity is categorized using semiquantitative systems dependent on pathologist description of staining [26, 47, 51] or percentage cells with staining [44]. The current study employed semiquantitative analyses that assessed subcellular location of staining and quantification by percentage of DAB-positive tissue. The results of the semiquantitative analyses revealed that the interobserver agreement was only fair. Therefore, the percentage DAB-positive tissue was used to analyze SSTR2 immunoreactivity instead. This type of analysis is only as reliable as the defined color spectrum cutoff for the presence or absence of staining. The program for this analysis was designed to be highly specific for positively stained tissue. This might have lowered the sensitivity for the identification of weakly positively stained tissue and favored identification of the strong membranous staining, which was typically more darkly stained than cytoplasmic staining. However, the latter could in fact be more appropriate because membranous staining is more heavily weighted when scored in many of the semiquantitative scoring systems; additional reassurance was provided by the fact that immunohistochemical analysis data exhibited strong correlation with gene expression data.

The entire acromegalic group expressed *DRD2* whereas *DRD2* expression is not found so consistently in human samples [46, 52, 53]; *PRL* expression was also detected in all samples. Therefore, the presence of *DRD2* might have been due to the presence of lactotrophs. In veterinary medicine, acromegalic cats undergo therapeutic total hypophysectomy rather than adenomectomy surgery, which might result in healthy pituitary tissue being adherent to the adenoma. Nevertheless, there was no correlation between *PRL* expression and *DRD2* expression, which argues against this and would be consistent with tumorous somatotroph DRD2 expression.

There was no difference in *DRD2* expression between acromegalic and control cats, although a moderate negative correlation between *DRD2* expression and pituitary size was detected. Dopamine has been shown to block cell cycle progression, and activation of *DRD2* by dopamine in a gastric cancer cell model has been shown to suppress cancer cell invasion [54, 55]. Additionally, the loss of *DRD2* in mice resulted in large prolactinomas [56]. *DRD2* loss in the pituitary might therefore also promote large somatotroph tumor formation in cats. These data also suggest that dopamine agonist therapy should be further evaluated in acromegalic cats and particularly in those with smaller pituitary tumors, because resistance to dopamine agonist therapy has been associated with lower *DRD2* expression in human GH-secreting adenomas [53].

One potential limitation to the study was that all cats with acromegaly were diabetic and receiving exogenous insulin. Previous studies in fish have shown SSTR expression to increase in a dose-dependent manner when exposed to increasing concentrations of insulin and glucose in the acute setting [57, 58]; whether this effect is sustained for >24 hours has not yet been reported. Our current studies found no correlations between SSTR expression and insulin dose or length of time the cat had been receiving exogenous insulin. Therefore, these findings suggest that chronic hyperglycemia or insulin therapy might not affect pituitary somatostatin receptor expression in cats.

In conclusion, the current study data reveal the heterogeneous expression of SSTRs in the pituitary gland from domestic cats without pituitary disease and those with acromegaly. Additionally, in parallel with human medicine, *DRD2* expression was correlated with pituitary tumor size in acromegalic cats. This study has revealed several parallels between humans and cats with acromegaly in terms of inhibitory receptor profiles. This receptor characterization aids our understanding of the morphology of the feline pituitary, and data suggest acromegalic cats as a model of the human disease in terms of developing therapeutics for GH inhibition.

## Abbreviations:

BSA: bovine serum albumin
CT: computed tomography
DAB: diaminobenzidine
HST: hypersomatotropism
IQR: interquartile range
PBS: phosphate buffered saline
PRL: prolactin
RT: room temperature
RVC: Royal Veterinary College
SSTR: somatostatin receptor
